# Video-assisted thoracoscopic surgery removal of a retained broken knife in the lung parenchyma: a case report and review of the literature

**DOI:** 10.1097/RC9.0000000000001108

**Published:** 2026-10-22

**Authors:** Ehsan Adib, Mohammad Vakili Ojarood

**Affiliations:** a Students Research Committee, School of Medicine, Ardabil University of Medical Sciences, Ardabil, Iran; b Cancer Immunology and Immunotherapy Research Center (CIIRC), Ardabil University of Medical Sciences, Ardabil, Iran; c Department of Surgery, School of Medicine, Ardabil University of Medical Sciences, Ardabil, Iran

**Keywords:** broken knife, case report, retained foreign body, VATS, video-assisted thoracoscopic surgery

## Abstract

**Background:**

Intrathoracic retained foreign bodies, such as knives, are typically removed via thoracotomy, which is an invasive procedure. Video-assisted thoracoscopic surgery (VATS) is a minimally invasive surgical procedure that can be a very good alternative to thoracotomy in selected patients. VATS is primarily used for the management and treatment of lung and pleural diseases, and VATS-assisted removal of intrathoracic retained foreign bodies has been reported in the limited literature to date.

**Case description:**

Our patient was a 32-year-old woman with no specific disease who, following a physical altercation, sustained multiple blows to the upper back with a fruit knife. With the last blow, the knife blade broke off from the handle and penetrated deep into the lung parenchyma in the right posterior hemithorax. The patient was referred to our hospital after 4 hours, and, considering the patient’s stable hemodynamics and multidisciplinary cooperation, it was decided to remove the retained knife blade via 3-port VATS. The operation lasted 45 minutes and was performed without any complications, and a knife blade measuring 10 × 1.2-cm was successfully removed from the posterior segment of the right lower lobe parenchyma without resection of the lung tissue. The patient was discharged without any complications on the ninth postoperative day, and subsequent follow-ups were uneventful.

**Discussion and conclusion:**

VATS as a minimally invasive technique can be a viable option as a primary approach in carefully selected, hemodynamically stable patients with accessible intrathoracic retained foreign bodies, when preoperative imaging does not reveal major cardiac or vascular injury, and when a suitably experienced center with immediate access to open conversion is available.

## Graphical abstract

https://links.lww.com/IJSCR/A194

## Background

For many years, thoracotomy has been the standard approach for exploring thoracic injuries because it allows complete visualization of the intrathoracic cavity, easy removal of foreign bodies under direct vision, control of bleeding, resection of damaged tissues, and expeditious repair of associated vascular or visceral injuries. Video-assisted thoracoscopic surgery (VATS), performed endoscopically through ports, is a minimally invasive technique compared with open surgery and can be a suitable alternative in some cases, with fewer surgery-related complications. It has become the standard of care for many lung and pleural diseases. The most common uses of VATS in trauma patients are the treatment of hemothorax and pneumothorax, and the evaluation of the diaphragm after injury. Another use, which has been described less frequently in the literature, is the removal of foreign bodies retained in the chest cavity^[^1–3^]^.

HIGHLIGHTS

- In hemodynamically stable patients requiring elective or emergency intrathoracic surgery, video-assisted thoracoscopic surgery (VATS) can be considered the first therapeutic approach.
- VATS is a minimally invasive surgical procedure suitable for removing various types of retained intrathoracic foreign bodies.
- Minimally invasive procedures, such as VATS, can be suitable alternatives to invasive procedures such as thoracotomy in various cases, depending on the discretion of the physician and the patient’s condition.
- In choosing a method for intrathoracic surgery, multidisciplinary collaboration and the patient's status should be considered when selecting the most appropriate surgical approach.

This case report was prepared in accordance with the principles of the Declaration of Helsinki and the SCARE guidelines^[^4^]^.

## Case presentation

A 32-year-old woman was referred from a hospital with limited facilities to our hospital’s emergency department due to a foreign body retained in the right posterior hemithorax, specifically a broken fruit-knife blade resulting from a physical altercation, embedded in a 3-centimeter (cm) penetrating laceration. She was transported to our hospital 4 hours after the initial injury. Upon initial examination, the patient presented with mild shortness of breath. Past medical, surgical, drug, and family histories were negative. On auscultation, decreased breath sounds were noted bilaterally, and the patient’s heart rhythm was regular without murmurs. Bleeding at the injury sites was controlled, with no active bleeding. No portion of the blade could be seen outside the patient’s posterior hemithorax. Two chest tubes were inserted on the right side, and one was inserted on the left side at the previous hospital.

She was alert and oriented, and her vital signs were within normal limits (temperature 36.5 °C, blood pressure 110/70 mmHg, respiratory rate 18 breaths per minute, heart rate 79 beats per minute, oxygen saturation 97% on room air, and GCS 15/15). The remainder of the physical examination was unremarkable. The results of blood tests, serological tests, and coagulation studies were within normal limits. An anteroposterior (AP) and lateral chest X-ray (CXR) revealed that the knife blade was still in situ at the level of the sixth intercostal space in the right posterior hemithorax, and contrast-enhanced computed tomography (CT) revealed evidence of atelectasis and mild pneumothorax in both lungs. There were no major vascular, cardiac, bronchial, mediastinal, or diaphragmatic injuries. The CT also confirmed the presence of a deeply embedded, long, hyperdense metallic foreign body in the posterior segment of the right lower-lobe lung parenchyma, with a sharp tip in a posterior-to-anteromedial position (Fig. 1).

**Figure 1.**
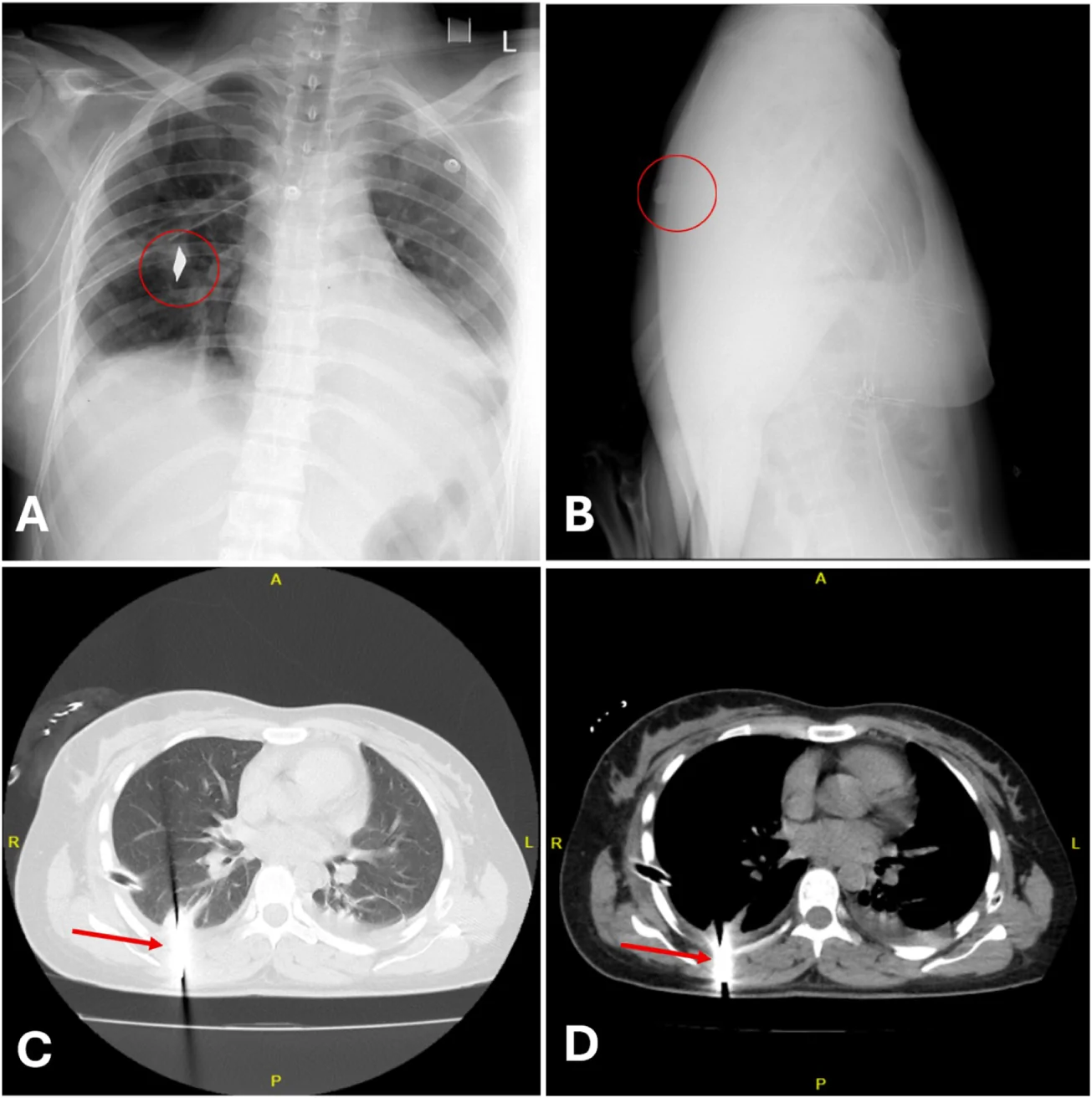
Chest X-ray revealed a metallic foreign body at the level of the sixth intercostal space in the posterior right hemithorax, in the posterior segment of the lower lobe parenchyma of the right lung, in a posterior-to-anteromedial position, which was a broken fruit knife blade (red circle; A and B), as well as a mild pneumothorax. CT-guided preoperative localization of the retained fruit knife blade in the posterior right hemithorax (red arrow; C and D).

It took 30 minutes from the moment the patient arrived at the hospital to the start of the operation. In the operating room (OR), C-arm imaging was performed to localize the position of the blade. An attempt was made by the thoracic surgeon to retrieve the blade with forceps at the wound site in the OR; however, this approach was halted due to the blade’s deep penetration and the high probability of bleeding and damage to adjacent structures. After a rapid intra-OR multidisciplinary meeting with the thoracic surgery, anesthesia, radiology, and pulmonology teams to determine whether to remove the blade via open surgery or VATS, it was ultimately decided to use 3-port VATS. However, the OR was set up for open surgery in case there was any difficulty removing the blade via VATS. After a preoperative work-up, the patient was placed in the right lateral position under general anesthesia with endotracheal intubation. The 30° camera (endoscope) and 10-mm port were inserted through the seventh intercostal space at the mid-axillary line, and then 5-mm ports were inserted through the sixth and eighth intercostal spaces. After the right lung was explored, a 10 × 1.2 cm blade with sharp ends was found to be mobile within the posterior segment of the right lower lobe parenchyma, allowing removal. The blade was manipulated such that it could be removed along its long axis through the port under direct thoracoscopic vision. The blade was grasped gently with thoracoscopic forceps and pulled slowly into the pleural space. While one end of the blade was still held with forceps, it was successfully removed en bloc from the pleural space through one of the ports without harming nearby structures or requiring lung wedge resection (Fig. 2).

**Figure 2.**
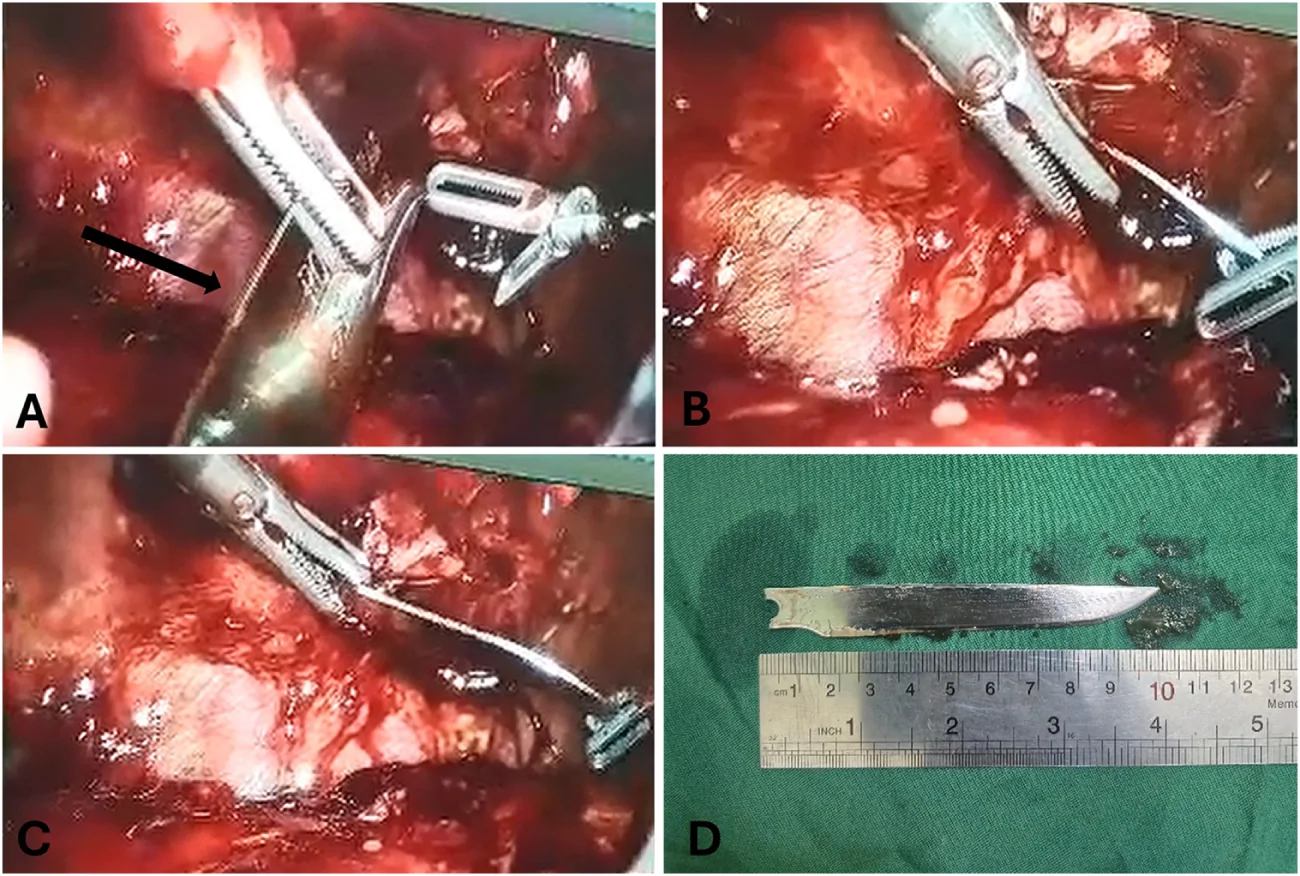
An intraoperative photograph showing a thoracoscopic view during retrieval of a knife blade (black arrow) that had penetrated deeply into the posterior segment of the right lower-lobe lung parenchyma, in a posterior-to-anteromedial position, and was grasped with forceps before removal (A–C). The 10 × 1.2-cm impaled knife blade after removal (D).

We found no ongoing bleeding or adhesions in the lung parenchyma, pleural space, or diaphragm on visual inspection, and no bleeding from the intercostal vessels or chest wall. The pleural and chest cavities were irrigated, the pulmonary laceration was repaired, and chest tubes were placed through the thoracoscopic ports and connected to an underwater drainage system. Postoperative irrigation and air leak testing confirmed that there were no complications. Intraoperative expansion of the lung lobes was confirmed at the end of the procedure. The total operating time (from incision to closure) was 45 minutes, and blood loss was less than 30 mL. She was transferred to the intensive care unit for close monitoring. There was no air leakage from the chest tubes, and postoperative CXR and CT scan on the third day after surgery revealed good lung expansion and no notable complications (Fig. 3). The chest drain was removed on postoperative day (POD) 4. On POD 5, the patient developed a mild fever and mild leukocytosis, which were attributed to a wound infection. This condition improved after 4 days of antibiotic therapy, and she was discharged from the hospital on POD 9 in good general condition, with analgesics, antibiotics, and tetanus prophylaxis. Her further recovery was uneventful, and she presented with no complaints at 1 and 4 months of follow-up (Graphical Abstract).

**Figure 3.**
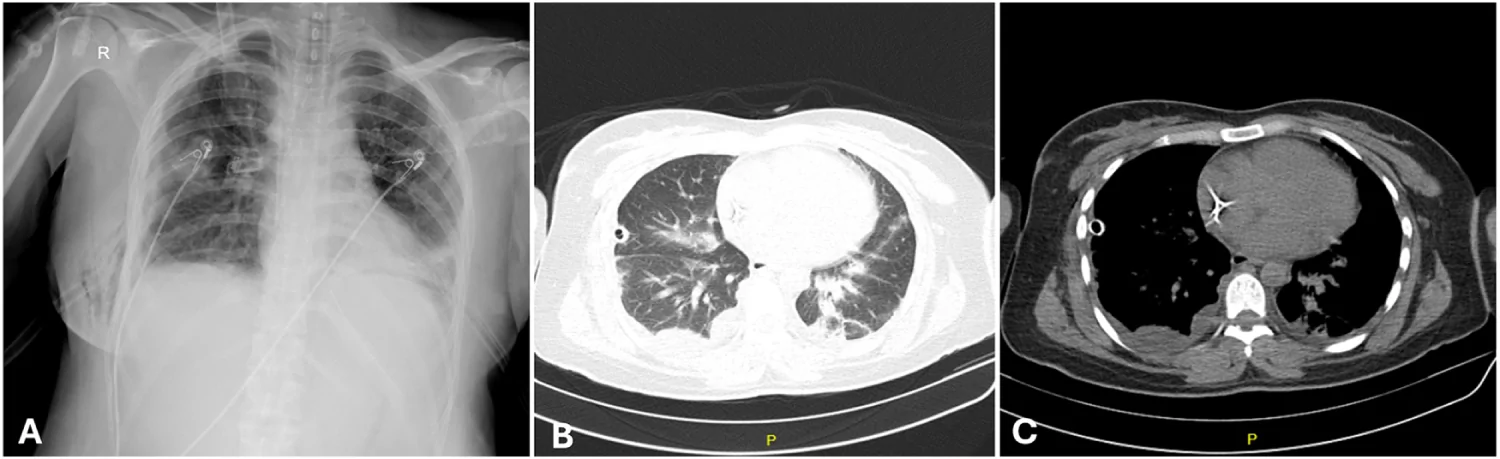
Immediate postoperative chest X-ray (A) and CT scans on the third day after surgery (B and C).

## Discussion

Penetrating trauma and aspiration of foreign bodies are important causes of foreign bodies remaining intrathoracically. Most of these foreign bodies are implanted in the lungs, bronchi, and esophagus, and rarely in the heart or pleura^[^5^]^. Retained metallic foreign bodies usually do not require removal in asymptomatic patients, and most surgeons recommend against removal because they are surrounded by fibrous tissue and become harmless over time. However, this decision should be based on an assessment and comparison of the risk of the foreign body remaining in the chest against the risks associated with attempting to remove it. However, removal is necessary for nonmetallic foreign bodies, as well as for large metallic objects or those with sharp edges ^[^1,6^]^.

Since the development of thoracoscopic surgery in the early 1900s, VATS has been considered a less invasive and more effective alternative to thoracotomy or laparotomy and is now the standard for the diagnosis and treatment of elective major thoracic surgery in hemodynamically stable patients, especially in the absence of great-vessel or cardiac injuries, with procedures performed under direct visualization, and several studies have demonstrated its feasibility and safety. VATS has become standard in many operations, such as lung and pleural biopsies, retained hemothorax, persistent pneumothorax, empyema, bulla resections, lobectomies, diagnosis of cardiac tamponade and the creation of a pericardial window, resection of mediastinal or bronchogenic cysts, blood clot evacuation, diaphragmatic exploration, and mass excision, especially for the treatment of early-stage lung cancer. Compared with thoracotomy, this approach has shown advantages, including milder postoperative pain, faster postoperative recovery, lower morbidity and mortality, less bleeding and transfusion volume, small incisions, a shorter duration of chest tube use, shorter hospitalization, early and safe return to work, reduced cost, and improved quality of life. However, the adoption and use of this technique are challenging because of the fulcrum effect, the loss of direct tactile sensation, the need for high operator experience, and the need to convert two-dimensional images into three-dimensional images. Additionally, the potential risk of intraoperative bleeding requires adequate management skills to prevent complications^[^1,2,7–9^]^.

A limited number of reports on the removal of intrathoracic foreign bodies using VATS, some of which have been published in recent years, are summarized in Table 1^[^1–3,5,6,9–32^]^. A comparison of similar cases in the literature (Table 1) revealed that the patients’ mean age was 40.9 years (range from 7 to 80), 23 % (*N* = 39, *n* = 9) of the patients were female, and 77% (*n* = 30) were male. Mechanisms of injury (*N* = 33) were mostly accidental events (*n* = 21), altercations (*n* = 7), and self-harm (*n* = 5). The common impaled objects (*N* = 39) were knives (in some cases only the blade and, in others, the blade with the handle; *n* = 9), bullets (*n* = 6), medical instruments (*n* = 5), and iron rods (*n* = 4). Therefore, our review highlights the high flexibility and ability of VATS to handle a wide array of impaled foreign bodies to meet the time-critical needs of severely injured patients.

**Table 1:** Patients with thoracic foreign body injury managed via VATS reported in the literature.

No/autor’s/publication date/country/cite	Age/sex	Mechanism of injury/cause of injury	Type of foreign body	Approximate size of the foreign object (mm)	Entry point/site of a foreign body	Sign and symptoms/operative or imaging findings	Time from injury to surgery (or hospital admission)	POD chest tube removal (day)	POD discharge (day)
1/our case/Iran	32/F	Altercation/NR	Retained fruit knife blade	100 × 12	Posterior segment of right lung lower lobe parenchyma	Mild shortness of breath/mild pneumothorax/bilaterally decreased lung sounds	4.5 h	4	9
2/Nakatsukasa *et al*/2025/Japan/^[^10^]^	80/M	Accidental event/accidental fish bone ingestion 5 years ago	Fish bone	40	Penetrated the esophageal wall and migrated into the lung	Upper abdominal pain and hemoptysis once or twice a year/small cavitary lesion and surrounding consolidation	5 years prior	1	9
3/Xu *et al*/2025/China/^[^11^]^	17/F	Accidental event/needle sticking while rolling on the bed	Sewing needle	23.3 × 2.8	Posterior basal segment of the right lower lung	Recurrent left-sided chest pain	9 years	1	2
4/Zhao *et al*/2023/China/^[^9^]^	63/M	Accidental event/falling from a height of 16 meters	T-shaped metallic bar	400	Traversing from the right supraclavicular region to the right fifth intercostal space close to the sternum, with nearly 30 cm of it penetrating the right thorax	Right chest pain/metallic bar was too long to fit inside the CT machine.	2 h	NR	14
5/Kakamad *et al*/2021/Iraq/^[^6^]^	46/M	Altercation/victim of two bullet entries	One bullet left	22	Inside the substance of the left lower lobe in the anterior segment	Vague pain in the left chest	26 years prior	NR	2
6/Aljehani *et al*/2019/Saudi Arabia/^[^12^]^	25/M	NR	Bullet	NR	Left nipple, around the 4th intercostal space, midclavicular line/left lower zone of lung	Mild dyspnea and pain in the back/fractured rib, left lower lobe lung contusion, and minimal hematoma and bleeding from the posterior segment of the lower lobe and hemothorax	NR	NR	NR
7/Danwang *et al*/2018/Cameroon/^[^13^]^	38/M	Altercation/NR	Knife	110	Left parascapular region midway between the scapula and the spine	Persistent left shoulder pain	7 weeks	NR	3
8/Dutta *et al*/2018/India/^[^14^]^	60/M	Accidental event/migrated the Kirschner wire from the acromioclavicular joint into the left lung	Kirschner wire	70	Traversing the left upper lobe of the lung and entering the mediastinum in the subaortic space	Cough and pain in the left chest	9 months	1	1
9/Liu *et al*/2018/Taiwan/^[^15^]^	54/M	Self-harm/history of schizophrenia	Jackknife with visible handle	60	Above the left nipple in the chest/penetrating through the mediastinum without injury to the heart and lung	Mediastinal hematoma	NR	NR	5
10/Liu *et al*/2018/Taiwan/^[^15^]^	47/M	Accidental event/fell two stories while working	Iron drill	NR	Right lower chest, below the nipple/seen at the lung base	Shortness of breath and right chest pain/right hemothorax, diaphragmatic and liver lacerations	NR	NR	7
11/Liu *et al*/2018/Taiwan/^[^15^]^	39/M	Accidental event/fell from a height	Cloth-wrapped iron rod	NR	The sixth intercostal space and midaxillary line/crossing the entire right chest cavity through the mediastinum	Hemopneumothorax, mediastinal hematoma	NR	NR	6
12/Liu *et al*/2018/Taiwan/^[^15^]^	44/M	Accidental event/angle grinder explosion while working	Angle grinder	NR	Right anterior chest	Mediastinal hematoma, pneumomediastinum, and pneumopericardium	NR	NR	14
13/Asaf *et al*/2017/India/^[^16^]^	7/M	Accidental event/accidental aspiration	Piece of magnet	10 × 5	Right hilar area	Severe cough with respiratory distress/subcutaneous emphysema in the neck	NR	NR	NR
14/Narasimhan *et al*/2017/India/^[^17^]^	80/M	Accidental event/breakage of the thoracentesis needle during thoracocentesis for pleural effusion	Thoracentesis needle	40	Embedded in the posterior parietal pleura and the chest wall, which was puncturing the diaphragm with respiratory movements.	Pleural effusion and pleural nodules (later revealed mesothelioma)	Immediately	1	NR
15/Narasimhan *et al*/2017/India/^[^17^]^	32/F	Accidental event/breakage of the thoracentesis needle during thoracocentesis for pleural effusion	Thoracentesis needle	40	Posterior-inferior part of the left pleural cavity with both ends embedded in the parietal pleura	Left-sided chest pain/pleural effusion and pleural nodules (later revealed tuberculous pleuritis)	1 week	1	NR
16/Ludwig *et al*/2017/Germany/^[^18^]^	31/M	Altercation/violent assault	Knife	NR	Left chest	Intercostal artery injury, left-sided	NR	NR	NR
						Hemopneumothorax, injury of the left upper lobe			
17/Peter *et al*/2016/China/^[^2^]^	27/M	NR	Cutter knife	55	Over the left lateral chest wall, close to the axilla/left upper lobe of the lung	Mild left pleural effusion	NR	2	2
18/Peter *et al*/2016/China/^[^2^]^	41/M	Accidental event/falling due to drunkenness	A fragment of the broken vase	10	Posterolateral left chest wall/over paraspinal soft tissue	Shock and desaturation/hemopneumothorax and subcutaneous emphysema	NR	3	3
19/Peter *et al*/2016/China/^[^2^]^	37/M	Self-harm/shooting himself multiple nails with a pressurized air stapler while drunk	Multiple nails	NR	Near the left nipple/nails penetrated the lingular segment and the lower lobe of the left lung	Intraparenchymal hematoma	NR	2	3
20/Ekeke *et al*/2016/USA/^[^5^]^	33/M	Self-harm/foreign body ingestion due to an extensive psychiatric condition	The writing pen migrated from the stomach to the pleural cavity	NR	Left apex of the pleural cavity	Left-sided pleuritic chest pain that radiated to the diaphragm/large left pleural effusion, and empyema	4 + 8 days	NR	NR
21/Shoko/2016/Japan/^[^19^]^	53/F	Accidental event/falling on glass	Two glass pieces	15 × 20 and 15 × 50	Left pleural cavity	Dyspnea and chest pain/hemopneumothorax	12 h	3	8
22/Lai *et al*/2016/Taiwan/^[^20^]^	30/M	Self-harm/NR	Chopstick	200	Left chest, between the heart and diaphragm	Pericardium laceration/trans-pericardial penetration without myocardial injury	NR	NR	NR
23/Yokosuka *et al*/2015/Japan/^[^22^]^	78/M	Accidental event/accidentally fell from the 3rd floor to the ground (a height of approximately 6 m)	2 metals reinforcement rods	Approximately 8 mm in diameter	One rod penetrated his right anterior chest, but did not exit through his back, and had penetrated the upper lobe of the lung. The other rod penetrated the left subclavicular space and exited through his back	Intrapulmonary hemorrhage and a large subcutaneous emphysema	NR	NR	10
24/Gupta *et al*/2014/India/^[^21^]^	60/M	Accidental event/needle breakage during thoracocentesis for pleural effusion	Broken thoracocentesis needle	80	Left pleural space and embedded in the chest wall	Left-sided chest pain, high-grade fever, and breathlessness/empyema	1 month	3	NR
25/Fujino *et al*/2012/Japan/^[^23^]^	55/F	Accidental event/swallowed a fish bone during dinner	Thai Fish Bone	22	A fish bone penetrated the esophagus into the right lower lobe, caudal-dorsal side of the right inferior pulmonary vein	Fever, chest pain	16 d	NR	NR
26/Khalil *et al*/2012/UK/^[^25^]^	20/M	Accidental event/accidental gunshot	Bullet	NR	Left of the lower sternal border/pericardial cavity	Asymptomatic	36 h	NR	4
27/Liu N *et al*/2011/USA/^[^24^]^	46/M	Accidental event/post revascularization (CABG)	Suture needle	26	Posterior-inferior intrapericardial position	Persistent left pleural effusion	2 months	1	2
28/Borgaonkar *et al*/2010/India/^[^26^]^	26/M	Accidental event/injury while working in a factory	Factory metal object	14-20 × 10	Right side of the chest at the nipple site/anterior segment of the right upper lobe	Chest pain and tachypnoea/lung laceration and hemothorax	NR	3	5
29/Dutta *et al*/2010/India/^[^27^]^	10/M	Accidental event/a flying metallic splinter originating from the blade of an electrical machine in a factory	Metallic splinter	26 × 5	Right lung parenchyma in the right lateral hemithorax	Chest pain and difficulty breathing/pleural effusion	2 days	5, 7	10
30/Komatsu *et al*/2009/Japan/^[^28^]^	57/F	Self-harm/history of schizophrenia	Kitchen knife	150	Right lower end of the sternum	Slightly decreased breath sounds at the right base/right hemothorax and partial tear of the pericardium	NR	NR	NR
31/Rückbeil *et al*/2009/Germany/^[^29^]^	69/F	Accidental event/transesophageal migrated mediastinal waved needle after eating strawberries, after MR-tomography	Waved needle	22	Placed in the sagittal direction above the bifurcation of the trachea between the esophagus and aortic arch	Dysphagia and retrosternal pain/decent hematoma	6 d	NR	6
32/Isenburg *et al*/2008/USA/^[^30^]^	37/F	NR	Long-bladed knife	NR	Left posterior chest/in the superior segment of the left lower lobe	Minimal bleeding and air leak from the wound, small amount of subcutaneous emphysema	20 + 15 min	2	1
33/Marsico *et al*/2008/Brazil/^[^1^]^	19/M	NR	Bullet	NR	Fourth right intercostal space, lateral to the nipple line/bullet located at the right cardiophrenic angle, free in the pleural cavity	Small ecchymosis in the middle lobe	NR	1	2
34/Marsico GA *et al*/2008/Brazil/^[^1^]^	25/M	NR	Bullet	NR	Fifth intercostal space, in the midaxillary line/bullet located in the paravertebral region (left costovertebral diaphragm recess), free in the pleural cavity	Small left hemothorax	NR	2	8
35/Edil *et al*/2007/USA/^[^31^]^	78/M	Accidental event/injury while using a brush-hog to clear land	Brush-hog blade	NR	Right posterior thorax, lateral to the scapular tip at the level of the eighth rib	Shortness of breath and pleuritic pain/hemothorax and pneumothorax	NR	1	2
36/Williams *et al*/2006/USA/^[^3^]^	23/M	Altercation/injured by a coworker with a nail gun while working	Carpentry nail	3-inch (76 mm)	Right chest, 1 cm lateral to the right nipple/nail head in the intercostal muscles of the right anterior-lateral chest wall, traverses the pleural space, penetrates the diaphragm, and enters the dome of the liver.	Chest pain/a small liver laceration	NR	NR	2
37/Williams *et al*/2006/USA/^[^3^]^	23/M	Altercation/NR	Retained knife blade	60	Right posterior axillary line at approximately the sixth intercostal space/the blade oriented in a posterior to the anteromedial position within the right chest/its tip was in the right lower lobe of the lung, and its base in the pleura at the site of the stab wound.	Right-sided chest pain and shortness of breath/Large right pneumothorax	NR	NR	7
38/Williams *et al*/2006/USA/^[^3^]^	29/F	NR	Bullet	NR	Left posterior trunk at approximately the level of the eighth thoracic vertebra/in the pleural space	Difficulty breathing/left hemothorax	NR	NR	3
39/Burack *et al*/2005/USA/^[^32^]^	25/M	Altercation/NR	Knife with visible handle	80	Left paraspinous region, midway between the tip of the scapula and the spine/intrathoracic position of the knife at the seventh thoracic vertebra, lateral to the mid descending thoracic aorta	Slightly decreased breath sounds on the left side/700 mL of blood and clot were evacuated from the posterior pleural cavity; injury of the superior segment, and hemorrhage from the lacerations	1 h	2	NR

M, male; F, female; POD, postoperative day; h, hour; NR, not reported; cm, centimeter.

## Conclusion

VATS as a minimally invasive technique can be a viable option as a primary approach in carefully selected, hemodynamically stable patients with accessible intrathoracic retained foreign bodies, when preoperative imaging does not reveal major cardiac or vascular injury and when a suitably experienced center with immediate access to open conversion is available.

## Data Availability

Not applicable.
